# Do alcohol use disorders impact on long term outcomes from intensive care?

**DOI:** 10.1186/s13054-015-0909-6

**Published:** 2015-04-22

**Authors:** Joanne M McPeake, Martin Shaw, Anna O’Neill, Ewan Forrest, Alex Puxty, Tara Quasim, John Kinsella

**Affiliations:** Glasgow Royal Infirmary, 84 Castle Street, Glasgow, G4 0SF UK; University of Glasgow, School of Medicine, Glasgow Royal Infirmary, New Lister Building, 10-16 Alexandra Parade, Glasgow, G31 2ER UK

## Abstract

**Introduction:**

There is limited evidence regarding the impact of alcohol use disorders on long term outcomes from intensive care. The aims of this study were to analyse the nature and complications of alcohol related admissions to intensive care and determine whether alcohol use disorders impact on survival at six months post ICU discharge.

**Method:**

This was an 18 month prospective observational cohort study in a 20 bedded mixed ICU, in a large teaching hospital in Scotland. On admission patients were allocated to one of three alcohol groups: low risk, harmful/hazardous, or alcohol dependency.

**Results:**

34.4% of patients were admitted with an alcohol use disorder. Those with an alcohol related admission (either harmful/hazardous or alcohol dependent) had an increased odds of developing septic shock during their admission, compared with the low risk group (OR 1.67; 95% CI 1.13-2.47, *p* = 0.01). After adjustment for all lifestyle factors which were significantly different between the groups, alcohol dependence was associated with more than a twofold increased odds of ICU mortality (OR 2.28; 95% CI 1.2-4.69, *p* = 0.01) and hospital mortality (OR 2.43; 95% CI 1.28-4.621, *p* = 0.004). After adjustment for deprivation category and age, alcohol dependence was associated with an almost two fold increased odds of mortality at six months post ICU discharge (HR 1.86; CI 1.30-2.70, *p* = 0.001).

**Conclusion:**

Alcohol use disorders are a significant risk factor for the development of septic shock in intensive care. Further, alcohol dependency is independently associated with poorer long term outcomes from intensive care.

**Electronic supplementary material:**

The online version of this article (doi:10.1186/s13054-015-0909-6) contains supplementary material, which is available to authorized users.

## Introduction

Alcohol misuse is a global problem compromising individual and social development. Globally, it contributes to approximately 3.3 million deaths per annum, resulting in 5.1% of the global burden of disease being attributable to alcohol [1]. Within the UK, Scotland has felt the societal and healthcare burden particularly severely [2]. Explanations for the problematic use of alcohol have focused on the effects of socioeconomic deprivation driven by the post-industrial decline that occurred in many British cities [3].

The impact that alcohol consumption has on the health of the Scottish Nation is startling, with one Scot dying every three hours of an alcohol attributable cause [4]. Scotland also has one of the fastest-growing rates of liver disease and cirrhosis in the world [2,5]; therefore, many patients admitted to hospital and the ICU will have a background of alcohol misuse.

Although there is an abundance of literature exploring the impact and burden of problematic alcohol use on public health and society, the impact that alcohol has in acute areas of healthcare services, such as the ICU, have not been well-described [6]. Given the controversial policies to reduce alcohol-related harm, such as minimum pricing, it is essential that clinicians and policy makers have information on all aspects of the harmful effects of alcohol.

Studies demonstrate that between 7 and 33% of ICU admissions are directly or indirectly related to alcohol [7-12]. This wide variation in incidence may be due to heterogeneity in the way alcohol-related admissions have been defined. The main focus of research interest has previously centred on severe alcohol dependency or alcoholism; however, it is now recognised that a spectrum of alcohol misuse categories exist [13]. Alcohol abuse, dependence and harmful alcohol use are now generally referred to as alcohol use disorders (AUDs) [14,15] and are recognised as different problems with individual treatment pathways.

AUDs can have widespread effects on the immune system and predispose abusers to increased risk of infection [16], sepsis and septic shock within critical care [9]. Furthermore, evidence suggests there are high healthcare utilisation costs in select groups such as patients admitted to ICU with alcohol withdrawal syndrome [17]. However, to date, only two studies report on the long-term outcomes of patients admitted to the ICU with an AUD. Christensen *et al*. (2012) conducted a prospective cohort study of 1,229 Swedish ICU patients, examining 30 day and three year mortality among alcoholic patients [18]. The 3-year mortality was 64.5% in the group of alcoholic patients with complications, compared to 40.9% among non-alcoholic patients. Due to their approach in assessing alcohol-related admissions, the researchers identified severely dependent patients only. This may have artificially inflated the reported mortality rates for the non-alcoholic patients and be the reason for the low levels of alcohol-related admissions seen in this study (7.3%) compared to previously cited research. Gacouin *et al*. (2014) explored whether at-risk drinking was independently associated with survival in non-trauma patients admitted to a French ICU in the year following ICU discharge. They also demonstrated significantly poorer outcomes at one year post ICU discharge in the at-risk drinkers group [12].

The aims of the present study were to prospectively analyse the nature and complications of alcohol-related admissions to critical care and determine whether AUDs were associated with survival at six months post ICU discharge within the UK critical care setting.

## Methods

An 18-month prospective cohort study was undertaken. Data collection took place between 1 June 2012 and the 31 December 2013. The study took place in a 20-bed mixed medical/surgical critical care unit in Glasgow Royal Infirmary (GRI). It is a tertiary referral centre for burn and pancreatic care and is situated in an area of high socioeconomic deprivation, with 42% of the most deprived geographical areas in Scotland residing in the GRI catchment area [19].

The primary inclusion criterion for the cohort study was all patients admitted to the ICU during the 18-month study period as level-three patients. The term level-three patients, refers to the UK Intensive Care Society definition of ICU patients. Level-three patients require multiple organ support or invasive respiratory support only [20]. Within the UK context, level-three patients require support within a critical care environment. The only exclusion was patients younger than 18 years. The majority of patients in Scottish ICUs are admitted under the Adults with Incapacity Act. Therefore, decisions about discharge would have been made by medical staff only, with patients unable to self-discharge.

Information on alcohol history was recorded by an admitting member of ICU medical staff. If an alcohol screen had been obtained in the ward setting or accident and emergency unit before admission to the ICU using the fast alcohol screening tool (FAST) score (Additional file 1: Table S1), this was used to assign the patient to the appropriate study group. If the FAST score had not been completed prior to ICU referral patients were classified using the World Health Organisation (WHO) classification. The admitting medical staff used a standardised flow sheet (Additional file 2: Table S2) to allocate patients to each category from the information available from relatives or the medical records. This sheet was available on the patient management system and additional hard copies were available. The study groups utilised were based on the WHO ICD-10 definition for AUDs [21] as low risk, harmful/hazardous or alcohol dependency (Additional file 2: Table S2). The alcohol groups used within the FAST screening tool are the same as the WHO groups.

Blood alcohol concentrations were not obtained as they have been shown to be less reliable than screening tools such as FAST and alcohol use disorder identification test (AUDIT) [22]. Patients were allocated to the smoking group if they were a current smoker. Furthermore, patients were allocated to the drug-use group in this study if they were admitted due to recreational drug use or were known intravenous drug users.

Data collected during the ICU stay was collected prospectively from various clinical information systems. The ICU utilises the Philips IntelliVue Clinical Information Portfolio (ICIP), locally known as CareVue (Revision D.03). CareVue is a repository of electronic patients’ notes, utilised for the duration of the patients’ ICU stay. Analysts from Information Services Division Scotland linked the ICU patient population being studied with the death registry for Scotland and extracted outcomes for patients at six months post ICU discharge.

Data obtained included: gender; age; acute physiology and chronic health evaluation (APACHE) II; ICU admission diagnosis; previous medical history including co-morbidities; baseline blood results; admitting speciality; days on ventilator; days on renal replacement therapy; diagnosis of septic shock; duration of vasopressor use; ICU outcome; total hospital stay; hospital outcome; six-month outcome and Scottish index of multiple deprivation (SIMD) decile. The SIMD is the Scottish Government’s official tool for identifying those geographical areas suffering from deprivation [19]. It incorporates different aspects of deprivation and summarises them into one score. Deprivation was defined as the two lowest deciles of the SIMD.

A patient was diagnosed with cirrhosis by an independent clinician outwith the research team. A diagnosis was made if they had features of chronic liver disease with evidence of portal hypertension, ascites, encephalopathy or liver and spleen imaging consistent with cirrhosis. Blood results did not guide this diagnosis. A diagnosis of septic shock was based on the Surviving Sepsis Campaign guidelines [23]. A definition of septic shock or cirrhosis could be made at any point during the ICU stay.

The West of Scotland Research Ethics approved this study (Reference Number 12/WS/0039). Individual patient consent was not required, as all data were collected as part of routine care.

### Statistics

The data were transferred to the statistical package RStudio (version 0.98.493) for statistical analysis [24]. All missing data fields were kept blank. The Kruskall-Wallis test and analysis of variance (ANOVA) were initially utilised to compare the three study groups. If there was a significant difference between the three study groups, a set of post hoc tests were carried out. Continuous variables were expressed as median and inter quartile range or mean and range and analysed using the Mann-Whitney *U*-test or the two sample *t*-test. Categorical variables were compared using the chi-squared test. At this point the Bonferroni correction was also used to adjust the error rate. All tests were two-sided and a *P*-value <0.05 was considered significant. Kaplan-Meier curves with the log rank test were used to compare six-month outcomes between the three study groups. Logistic regression models were used to determine independent associations between variables and a Cox proportion model was used to determine the difference between the three study groups with survival analysis. These results were expressed in terms of the odds ratio (OR) and the hazard ratio (HR) with the corresponding 95% CI.

There are many approaches to building and creating statistical models in medical research [25]. Within this study, models were ranked using the Akaike information criterion (AIC). Although this approach was taken to determine the best available model for the data, clinical relevance and applicability was also used to ensure that the statistical models being created would be clinically meaningful.

## Results

**Figure 1:**
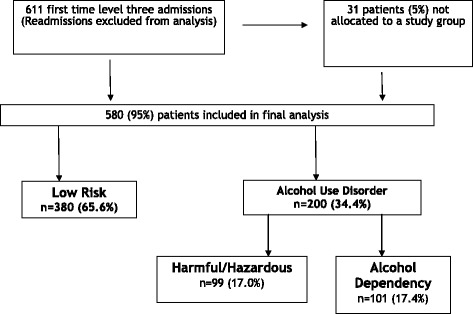
Included and excluded participants in the 18-month prospective cohort study.

During the 18 month study period, 611 level-three patients were admitted to the ICU. Of these, 31 patients were not allocated to any study group in their medical notes due to lack of information on previous alcohol use from the patients, relatives or the medical records. These patients were excluded from the study leaving 580 patients with a classification of their alcohol use (Figure 1).

Of the 580 patients evaluated, 380 (65.6%) were in the low-risk group, 99 (17.0%) were in the harmful/hazardous group and the remaining 101 (17.4%) patients were in the alcohol dependency group (Figure 1). A breakdown of the different clinical variables analysed and the differences in these variables across the three study groups is given in Additional file 3: Table S3. Additional file 4: Table S4 details the admitting area and speciality for every patient.

### Demographic data

The mean age of patients admitted to the unit during the study period was 57 years (range 19 to 90), with a significant differences in age seen between the three study groups (*P* <0.001). There was also a significant difference in smoking and drug use amongst the three study groups (*P* <0.001). Similarly, the number of patients admitted with a background of drug misuse was significantly higher in the alcohol-related groups (*P* <0.001). Furthermore, patients who were admitted with a background of drug and alcohol misuse had more than fourfold increased odds of living in the two lowest deciles of the SIMD (OR 4.89, 95% CI 2.51, 10.46; *P* <0.001).

### Interventions

During the ICU stay 549 (94.7%) patients were mechanically ventilated with no significant difference between the three study groups (*P* = 0.134) in the duration of mechanical ventilation (Additional file 5: Table S3). There was a significant difference in the use of vasopressor therapy between the low-risk and harmful/hazardous group (59.2% versus 43.3%, *P* = 0.02) and between the harmful/hazardous and alcohol dependent groups (43.3% versus 58.4%, vs. = 0.014). The median number of days in which patients required vasopressor therapy was also significantly different between the three groups (Additional file 3: Table S3) with the alcohol-dependency group requiring vasopressor support for longer than those admitted in the low-risk group (2 days versus 3 days; *P* = 0.042).

### Septic shock

Overall, 139 (24%) patients had a diagnosis of septic shock: 20.5% of patients in the low-risk group developed septic shock in comparison to 32.2% in the harmful/hazardous group and 28.7% in the alcohol-dependent group. In simple logistic regression those patients who were admitted with an AUD had an increased odds of developing septic shock during their ICU admission compared with the low-risk group (OR 1.67, 95% CI 1.13, 2.47, *P* = 0.0098).

### ICU Length of stay (LOS)

**Figure 2:**
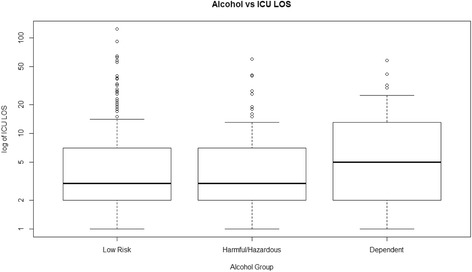
Boxplot comparing median ICU length of stay (LOS) between the three study groups.

Median ICU LOS was significantly different between the study groups (Figure 2). A log transformation highlighted the relative differences in ICU LOS that were compared. After the Bonferroni correction had been applied, only the difference between the harmful/hazardous and alcohol-dependency group remained significant (*P* = 0.013).

### ICU outcome

On univariate analysis there was no difference in ICU outcome between the three groups (*P* = 0.15). After adjustment for all lifestyle factors that were significantly different between the groups (age, smoking and drug use), alcohol dependence was associated with more than a twofold increased odds of ICU mortality (OR 2.28, 95% CI 1.2, 4.69, *P* = 0.012). There was no significant difference in ICU outcome for those patients with liver cirrhosis compared to those patients admitted without liver cirrhosis (*P* = 0.1877). Despite evidence suggesting a higher rate of mortality for those patients admitted with burns and alcohol dependency, there was no difference in ICU outcome between those patients admitted from burns and plastics with and AUD and the general ICU population in this study (25 versus 25%).

### Hospital outcome

On univariate analysis there was a significant difference in hospital outcome between the three study groups (*P* = 0.044) (Additional file 3: Table S3) but this difference did not remain significant after correction for multiple tests. After adjustment for lifestyle factors, alcohol dependence was associated with more than a twofold increased odds of hospital mortality (OR 2.43, 95% CI 1.28, 4.62, *P* = 0.004).

### Six-month outcome

**Figure 3:**
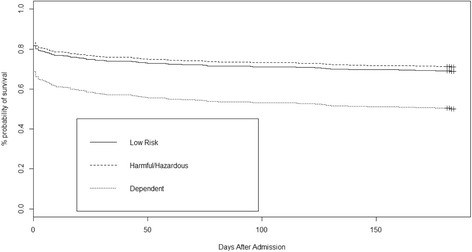
Kaplan-Meier curve for patients from the three study groups at 6 months post ICU discharge (adjusted for the presence of deprivation and age).

Six-month post ICU discharge mortality in this cohort of patients was 37%. With univariate analysis there was no difference in outcome between the three study groups. However, after adjustment for deprivation category (living in the two lowest deciles of the SIMD) and age, alcohol dependence was associated with an almost twofold increased odds of mortality at six months post ICU discharge (HR 1.86, CI 1.30, 2.70, *P* = 0.001) (Additional file 5: Table S5). A log rank test was performed on the stratified Cox proportional hazards model, which demonstrated the influence the model had on survival (*P* <0.001) (Figure 3).

## Discussion

In this study a high proportion of patients had AUDs. Compared to other ICU admissions, they were younger, lived in areas of higher socioeconomic deprivation, were more likely to take drugs and smoke and had an increased odds of developing septic shock. Furthermore, after adjusting for age and deprivation, alcohol dependence was associated with an almost twofold increased odds of mortality at six months post ICU discharge.

The proportion of alcohol-related admissions (34.4%) reported in this study is similar to that in a recent French study [12]; however, this number is higher than a previous snapshot audit undertaken in Scotland, which estimated that 25.4% of ICU admissions were related to alcohol [11]. This difference may reflect the deprived geographical area where the study centre sits; there is strong body of evidence demonstrating the link between the deprivation gradient, alcoholism and alcohol-related deaths [26]. As a tertiary referral centre for pancreatitis and burns we know that alcohol is also a significant risk factor in being admitted with these diagnoses.

In this study, patients admitted with alcohol dependency required vasopressor support for significantly longer than those admitted in the low-risk group. This may be due to a greater use of sedative agents to manage alcohol withdrawal, as well as the finding that patients with an alcohol-related admission had almost twofold increased odds of developing septic shock during their ICU admission [27,28]. Immunological and non-immunological factors may contribute to increased susceptibility to infection in patients with chronic alcohol exposure [16]. For example, animal and human studies have demonstrated that chronic alcohol consumption may inhibit the production of important cytokines [29], modify neutrophil functions and suppress T-cell-mediated immunity [30]. These cellular changes could lead to an increased predilection to infection which may contribute to systemic problems [9].

The poor ICU and hospital outcomes in patients with alcohol dependency seen in this study are also consistent with the literature [9,12]. Interestingly, patients with liver cirrhosis did not have a significantly worse outcome than those patients without liver cirrhosis. This demonstrates that it is the presence of alcohol dependency in patients admitted to ICU that influences poor ICU outcomes, rather than significant alcohol-related comorbidities. The long-term outcome results generated in this study reflect the results of two other European papers on this topic, which demonstrated the negative impact of AUDs on longer-term outcomes from critical care [12,18]. The poor long-term outcomes seen in this cohort may be due to the social problems that many of these patients may face after discharge from critical care. Additionally, these patients are more likely to develop septic shock when in ICU, which is known to impact on long-term mortality [31]. As far as we can establish this is the first British study that has explored longe-term outcomes in this patient cohort.

Providing ICU care is expensive and therefore it is vital that those who survive are given the support they require during recovery from critical illness. Some recent studies have suggested that early intervention within the ICU environment in the form of brief interventions may be beneficial, and an ICU admission may represent a teachable moment for patients with an AUD [32]. Much of this research was undertaken with patients during the ICU stay, which may not give a full picture of the multifaceted interventions required for this cohort in the longer term [15]. More research, including pathways of optimal rehabilitation, is required in this area.

### Study limitations

The prospective approach to assessing patients does have strengths, however, AUDs could have been misclassified in this study. Appropriate assessment and evaluation of this patient cohort is challenging due to limited communication and interaction [33]. Validated scoring tools for the assessment of AUDs (that is the FAST or AUDIT) [13,34] have not been extensively validated in the non-verbal ICU population [35]. Whilst in some clinical settings they have been validated for use by proxies, there appears to be no study that has validated this approach within the European critical care setting [36]. As a result they are rarely used in the critical care setting in the UK [36]. More research exploring the assessment of AUDs in the critical care setting in required.

This single-centre study was undertaken in an area of high deprivation and where alcohol-related illness is a significant public health issue. Therefore, the high numbers of patients with AUDs identified may not be representative of all ICUs. However, this paper does offer a unique insight into the impact of alcohol and its link with deprivation in the critical care setting.

Last, we did not explore delirium or alcohol withdrawal during the ICU stay in this cohort. Alcohol is a known risk factor for the development of delirium and delirium is a known risk factor for poor long-term outcomes. An area for future research would be to explore the relative contribution of alcohol dependency and alcohol withdrawal and their impact on long-term outcomes from critical care.

## Conclusion

In conclusion, this prospective observational study has demonstrated that alcohol-related admissions represent a significant issue to the ICU environment. It has explored the nature and complications of AUDs in the ICU and hospital environment and has demonstrated their negative impact on ICU survival up to six months post ICU discharge.

## Key messages

- Patients with alcohol use disorders represent a significant proportion of admissions to ICU.
- Patients admitted to ICU with an alcohol use disorder are vulnerable to various complications
- After adjustment for other lifestyle factors, patients with a background of alcohol dependency have significantly worse ICU and hospital outcomes in comparison to those without an alcohol use disorder;
- After adjustment, patients with a background of alcohol dependency have worse long-term outcomes from ICU compared to patients without an alcohol use disorder.

## Additional files

Additional file 1:
**Fast alcohol screening tool (FAST).**
Additional file 2:
**Definitions of alcohol use disorders (adapted from World Health Organisation 2010).**
Additional file 3:
**Differences in clinical variables between the three study groups.**
Additional file 4:
**Admitting speciality and area.**
Additional file 5:
**Risk-adjusted association between alcohol dependence and six month outcome.**

## Abbreviations

AIC: Akaike’s information criterion
APACHE II: acute physiology and chronic health evaluation
AUD: alcohol use disorder
AUDIT: alcohol use disorder identification test
FAST: fast alcohol screening tool
GRI: Glasgow Royal Infirmary
HR: hazard ratio
LOS: length of stay
OR: odds ratio
SIMD: Scottish index of multiple deprivation
WHO: World Health Organisation
