# Supplementary material for: Do alcohol use disorders impact on long term outcomes from intensive care?
Source: Crit Care. 2015 Apr 22;19(1):185. doi: 10.1186/s13054-015-0909-6 (PMC4440292; doi:10.1186/s13054-015-0909-6)
Supplement: Additional file 1: — Fast alcohol screening tool (FAST). [file 13054_2015_909_MOESM1_ESM.docx]

| **FAST** | **Scoring system** | | | | | **Your score** |
| --- | --- | --- | --- | --- | --- | --- |
|  | **0** | **1** | **2** | **3** | **4** |  |
| How often have you had 6 or more units if female, or 8 or more if male, on a single occasion in the last year? | Never | Less than monthly | Monthly | Weekly | Daily or almost daily |  |
| **Only answer the following questions if the answer above is Never (0), Less than monthly (1) or Monthly (2). Stop here if the answer is Weekly (3) or Daily (4).** | | | | | | |
| How often during the last year have you failed to do what was normally expected from you because of your drinking? | Never | Less than monthly | Monthly | Weekly | Daily or almost daily |  |
| How often during the last year have you been unable to remember what happened the night before because you had been drinking? | Never | Less than monthly | Monthly | Weekly | Daily or almost daily |  |
| Has a relative or friend, doctor or other health worker been concerned about your drinking or suggested that you cut down? | No |  | Yes, but not in the last year |  | Yes, during the last year |  |

**Table One: The FAST screening tool (Hodgson et al 2002)**

**Scoring:**

If the score is 0, 1 or 2 on the first question continue with the next three questions

If score is 3 or 4 on the first question – stop here.

**An overall total score of 3 or more is FAST positive**.

**Next Step:**

If FAST positive, complete remaining AUDIT questions (this may include the three remaining questions above as well as the other six questions) to obtain a full AUDIT score.

**Scoring from the AUDIT score:**

0-7: Indicates sensible drinking

8-15: Indicates hazardous drinking

16-19: Indicates harmful drinking

20+ Indicates possible dependence
