# Supplementary material for: Do alcohol use disorders impact on long term outcomes from intensive care?
Source: Crit Care. 2015 Apr 22;19(1):185. doi: 10.1186/s13054-015-0909-6 (PMC4440292; doi:10.1186/s13054-015-0909-6)
Supplement: Additional file 2: — Definitions of alcohol use disorders (adapted from World Health Organisation 2010). [file 13054_2015_909_MOESM2_ESM.docx]

**Table Two. Definitions of Alcohol Use Disorders (Adapted from WHO 2010)**

**No Risk/Low risk:**

Individuals who:

- Consume no alcohol;
- Have experienced no or minimal harm as a result of alcohol use.

**Harmful Use:**

- Alcohol is responsible for or has substantially contributed to physical or psychological harm, including impaired judgement or dysfunctional behaviour ;
- The nature of harm is clearly identifiable (i.e. falls/ absence from work);
- The pattern has persisted for at least one month previous to admission or has occurred repeatedly within a 12 month period.

**Alcohol Dependence (should be made if three or more of the following are present):**

- A strong desire or sense of compulsion to take alcohol;
- Difficulty in controlling drinking in terms of: onset, termination or level of use;
- A physiological *withdrawal* state is present when drinking has ceased or been reduced;
- Drinking to relieve or avoid withdrawal symptoms;
- Evidence of *tolerance*, such that increased doses of alcohol are required in order to achieve effects originally produced by lower amounts (examples are when individuals take daily doses sufficient to incapacitate or severely hurt non-tolerant users);
- Preoccupation with alcohol use to the detriment of other interests (e.g. social or occupational)
- Persistent alcohol use despite awareness of harmful consequences, such as physical harm (liver impairment), depressive mood states consequent to periods of heavy drinking, or alcohol related impairment of cognitive function.
