# Supplementary material for: Do alcohol use disorders impact on long term outcomes from intensive care?
Source: Crit Care. 2015 Apr 22;19(1):185. doi: 10.1186/s13054-015-0909-6 (PMC4440292; doi:10.1186/s13054-015-0909-6)
Supplement: Additional file 3: — Differences in clinical variables between the three study groups. [file 13054_2015_909_MOESM3_ESM.docx]

|  | | | | |
| --- | --- | --- | --- | --- |
| **Characteristics Low Risk Harmful/Hazardous Alcohol Dependency**  **n=380 (65.6%) n=99(17.0%) n=101(17.4%)  *p* value** | | | | |
| **Baseline Demographics** | | | | |
| Age, Mean (Range) | 61.0 (19-90) | 50.3 (19-81) | 48.9 (27-76) | **<0.001** |
| Gender (Male) | 186 (48.9%) | 77 (77.8%) | 76 (75.2%) | **<0.001** |
| APACHE II, Mean (Range) | 20.6 (2-50) | 20.2 (3-41) | 22 (8-47) | 0.22 |
| Known socio economic deprivation (2 lowest deciles of SIMD) | 178 (46.8%) | 64 (64.6%) | 65 (64.4%) | **<0.001** |
| Days in Hospital Pre ICU admission, Median (IQR) | 1 (0-4) | 0 (0-1) | 1 (0-3) | **<0.001** |
| Smoking | 117 (30.1%) | 52 (52.5%) | 61 (60.4%) | **<0.001** |
| Drug Use | 13 (3.4%) | 23 (23.2%) | 22 (21.8%) | **<0.001** |
| Liver Cirrhosis  (Alcohol or non alcohol related) | 14 (3.7%) | 6(6.1%) | 55 (54.5%) | **<0.001** |
| **ICU Admission** | | | | |
| ICU Length of Stay, Median (IQR) | 3 (2-7) | 3 (2-7) | 5 (2-13) | **0.009** |
| Ventilator Days, Median (IQR) | 2 (2-6) | 2 (1.5-4) | 3 (2-9) | 0.134 |
| Vasopressor Therapy Used | 225 (59.2%) | 43 (43.3%) | 59 (58.4%) | **0.017** |
| Vasopressor Days, Median (IQR) | 2 (2-4) | 2(2-4) | 3(0-4) | **0.0475** |
| RRT Therapy used | 64 (16.8%) | 10 (10.1%) | 19 (18.8%) | 0.1872 |
| RRT Days, Median (IQR) | 3 (1-27) | 5(2.25-10.5) | 4(1-6) | 0.5944 |
| Diagnosis of Septic Shock | 78 (20.5%) | 32 (32.2%) | 29 (28.7%) | **0.029** |
| ICU Non Survivor | 98 (16.9%) | 18 (18.2%) | 30 (29.7%) | 0.15 |
| Readmission to the ICU | 37 (9.7%) | 8(8%) | 11 (10.9%) | 0.79 |
| **Post ICU** | | | | |
| Days in Hospital Post ICU, Median (IQR) | 14 (7-33) | 7 (2-20) | 14 (6-26) | **<0.001** |
| Total Hospital stay, Median (IQR) | 18 (8-38) | 9 (4-24.5) | 19 (7-39) | **<0.001** |
| Non Survivor (Hospital) | 128 (33.6%) | 22 (22.2%) | 38 (37.5%) | **0.044** |
| **Long Term Outcomes** | | | | |
| Discharged to long term rehabilitation | 19(5%) | 6(6.1%) | 3(3%) | 0.1866 |
| Non Survivor (6 months) | 145 (38.2%) | 26 (26.3%) | 44(43.6%) | 0.5105 |

**Table Three: Differences in clinical variables between the three study groups**
