# Supplementary material for: Do alcohol use disorders impact on long term outcomes from intensive care?
Source: Crit Care. 2015 Apr 22;19(1):185. doi: 10.1186/s13054-015-0909-6 (PMC4440292; doi:10.1186/s13054-015-0909-6)
Supplement: Additional file 4: — Admitting speciality and area. [file 13054_2015_909_MOESM4_ESM.docx]

| **Admitting Speciality/ Admitting Area** | **Low Risk**  **n=380 (65.6%)** | **Harmful/ Hazardous**  **n=99**  **(17.0%)** | | **Alcohol Dependency**  **n=101**  **(17.4%)** | ***p* value** |
| --- | --- | --- | --- | --- | --- |
| **Admitting Speciality** |  | |  |  |  |
| Respiratory Medicine | 71 (19%) | | 15(15.2%) | 14(13.8%) |  |
| Gastroenterology | 7 (2%) | | 1(1%) | 23(22.8%) |  |
| General Surgery | 143 (38%) | | 26(26.3%) | 22(21.8%) |  |
| Burns and Plastics | 32 (8%) | | 7(7%) | 5(4.9%) |  |
| Orthopaedics | 10 (3%) | | 4(4%) | 4(4%) |  |
| Cardiology | 28(7%) | | 5(5%) | 3(3%) |  |
| General Medicine | 32 (8%) | | 31(31.3%) | 19(18.8%) |  |
| Gynaecology/Obstetrics | 21 (5.5%) | | 0 | 0 |  |
| ENT | 2 (0.5%) | | 0 | 0 |  |
| Neurology/Neurosurgical | 32 (8%) | | 10(10.1%) | 11(10.9%) |  |
| Vascular | 1 (0.3%) | | 0 | 0 |  |
| Urology | 1 (0.3%) | | 0 | 0 | **<0.001** |
| **Area Admitted from** |  | |  |  |  |
| Ward in hospital (GRI) | 127 (33.4%) | | 26(26.3%) | 41(40.6%) |  |
| Accident and Emergency | 89(23.4%) | | 45(45.4%) | 39(38.6%) |  |
| Theatre/Recovery | 123(32.3%) | | 16(16.2%) | 11(10.9%) |  |
| External Transfer from  other hospital | 41(10.8%) | | 12(12.1%) | 10(9.9%) | **<0.001** |

**Table 4. Admitting speciality and admitting area**
