# Supplementary material for: Do alcohol use disorders impact on long term outcomes from intensive care?
Source: Crit Care. 2015 Apr 22;19(1):185. doi: 10.1186/s13054-015-0909-6 (PMC4440292; doi:10.1186/s13054-015-0909-6)
Supplement: Additional file 5: — Risk-adjusted association between alcohol dependence and six month outcome. [file 13054_2015_909_MOESM5_ESM.docx]

**Table 5. Risk adjusted association between alcohol dependence and six month outcome**

| **Variable Adjusted Hazard Ratio (95% CI)** | |
| --- | --- |
| Alcohol Dependence | 1.86 (1.30-2.70) |
| Age | 1.03 (1.02-1.05) |
| Deprivation | 1.11 (0.84-1.45) |
